# Global Diversification at the Harsh Sea-Land Interface: Mitochondrial Phylogeny of the Supralittoral Isopod Genus *Tylos* (Tylidae, Oniscidea)

**DOI:** 10.1371/journal.pone.0094081

**Published:** 2014-04-15

**Authors:** Luis A. Hurtado, Eun J. Lee, Mariana Mateos, Stefano Taiti

**Affiliations:** 1 Department of Wildlife and Fisheries Sciences, Texas A&M University, College Station, Texas, United States of America; 2 Istituto per lo Studio degli Ecosistemi, CNR, Florence, Italy; University of Poitiers, France

## Abstract

The supralittoral environment, at the transition between sea and land, is characterized by harsh conditions for life. Nonetheless, evolution of terrestrial isopods (Oniscidea), the only group of Crustacea fully adapted to live on land, appears to have involved a transitional step within the supralittoral. The two most basal oniscidean lineages (Ligiidae and Tylidae) have representatives that successfully colonized the supralittoral. One of them is the genus *Tylos*, which is found exclusively in supralittoral sandy beaches from tropical and subtropical coasts around the world. Comprehensive phylogenetic hypotheses for this genus are lacking, which are necessary for understanding the evolution and biogeography of a lineage that successfully diversified in the harsh sea-land interface. Herein, we studied the phylogenetic relationships among 17 of the 21 currently recognized species of the genus *Tylos*, based on sequences from four mitochondrial genes (Cytochrome Oxidase I, Cytochrome b, 16S rDNA, and 12S rDNA). Maximum Likelihood and Bayesian phylogenetic analyses identified several lineages with deep divergences and discrete geographic distributions. Phylogenetic and distributional patterns of *Tylos* provide important clues on the biogeography and evolution of this group. Large divergences among the most basal clades are consistent with ancient splits. Due to the biological characteristics of *Tylos*, which likely prevent dispersal of these isopods across vast oceanic scales, we argue that tectonic events rather than trans-oceanic dispersal explain the distribution of *Tylos* in different continents. Overwater dispersal, however, likely enabled range expansions within some basins, and explains the colonization of volcanic oceanic islands. Present-day distributions were also likely influenced by sea level and climate changes. High levels of allopatric cryptic genetic differentiation are observed in different regions of the world, implying that the dispersal abilities of *Tylos* isopods are more limited than previously thought. Our results indicate that a taxonomic revision of this group is necessary.

## Introduction

The supralittoral, at the transition between sea and land, comprises a very narrow vertical stretch of the shoreline characterized by harsh conditions for life, such as regular exposure to extreme temperatures, wind, wave splash, storm surge, rapid and extreme changes in salinity (e.g. high salinity due to evaporation during low tide and low salinity due to fresh water input from rain), and predation by terrestrial animals and seabirds [1]. Adaptation to completing the life cycle within this harsh environment is considered a crucial step in the evolution of Oniscidea [2], a speciose group of isopods (ca. 3700 species) that has colonized almost every terrestrial habitat [3], representing the only group of Crustacea fully adapted to live on land. Isopod colonization of land from the sea appears to have involved a transitional step within the supralittoral. The two most basal oniscidean lineages, Ligiidae and Tylidae [4], [5], successfully colonized the supralittoral. Within Ligiidae, the genus *Ligia* Fabricius, 1798 occurs in rocky supralittoral habitats around the world, with only a few species adapted to live inland. Evolution of terrestrial oniscideans from ancestral marine isopods is proposed to have proceeded from a *Ligia*-like ancestor [2]. Within Tylidae, the genus *Tylos* Audouin, 1826 is comprised entirely of supralittoral species distributed mainly in tropical and subtropical sandy beaches throughout the world [6], [7]. Regional phylogeographical studies of members of *Ligia* and *Tylos* have revealed high levels of isolation and phylogeographic structure at small geographic scales, consistent with biological characteristics that confer limited vagility to these isopods [8]–[10]. The lack of comprehensive phylogenetic hypotheses for these two genera, however, hampers our understanding on the evolution of two groups that successfully diversified in the vast, yet harsh, sea-land interface.

Isopods of the genus *Tylos* occur in the upper intertidal on sand, mud, in cracks and crevices, and under algal detritus or rocks [6]. *Tylos* currently contains 21 accepted species, with many more originally described, but subsequently synonymized [11], [12]. Many specimens from different regions in the world were incorrectly assigned to *T. latreillii* Audouin, 1826, which was originally described from an unspecified location in Egypt [13], but currently considered a *nomen dubium* [14]. This taxonomic confusion has likely contributed to the misconception that members of *Tylos* are highly vagile (e.g. [15]), a notion that is at odds with their biological characteristics, which suggest very restricted dispersal potential. As all peracarids, *Tylos* isopods lack a planktonic larval stage (i.e., they are direct developers). In addition, adults actively avoid entering the water, where they are unable to survive submerged beyond a few hours, and have extremely limited swimming abilities [6], [7]. Juveniles of some species, however, may be able to surf by rolling themselves into a ball, potentially facilitating dispersal among nearby beaches [7], [15]. Consistent with their biological characteristics, high levels of population genetic differentiation have been observed at small geographic scales in members of *Tylos*, implying that surrounding unsuitable habitats constitute effective dispersal barriers and that the potential for population isolation is high [9]. Furthermore, factors that can dramatically modify the distribution of coastline habitats, such as tectonic activity and eustatic sea level fluctuations, as well as climate change, appear to strongly influence the evolutionary histories of these isopods [9].

Herein, we inferred a mitochondrial phylogeny of the genus *Tylos*, by examination of 17 of the 21 currently recognized species. The results provide insights into the evolutionary history and biogeography of a group that successfully diversified within the harsh sea-land interface. This represents, to our knowledge, the first study to examine the relationships of a supralittoral endemic taxon at a global scale.

## Methods

### 2.1 Sampling and Molecular Methods

**Figure 1:**
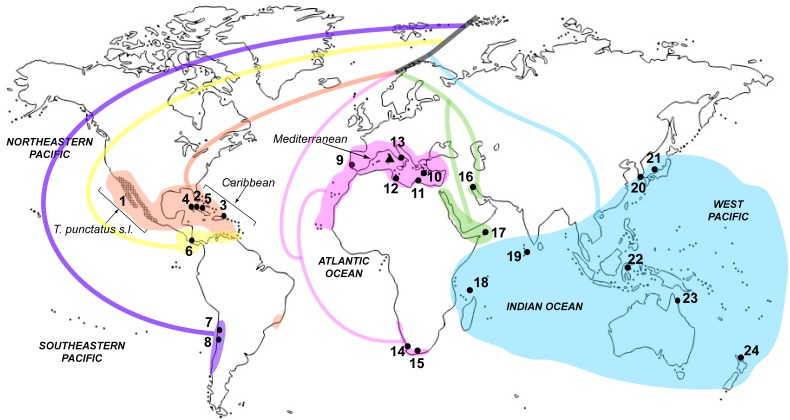
Sampling localities, approximate distribution ranges of clades, and inferred relationships. Major clades and their approximate distribution ranges are distinguished by different colors (see Results and Fig. 2). Phylogenetic relationships based on Fig. 2. Numbers correspond to sample IDs shown in Fig. 2 and Table S1.

We obtained tissue samples from 16 of the 21 currently recognized species of *Tylos* (Fig. 1) and used published sequences from one additional valid species from California, *T. punctatus* Holmes & Gay, 1909, and its close relatives, with which it forms *T. punctatus* sensu lato from California to the western coast of Mexico [9]. Most of the samples were obtained from the Museo di Storia Naturale “La Specola”, Zoological section, in Florence, Italy (MZUF); other researchers and museums kindly provided the remaining samples (Table S1). The sample from Puerto Rico, for which no specific permissions were required, was collected by LAH. None of the fieldwork involved endangered or protected species. Photographs of the ventral plates of the fifth pleonite, regarded as a species-diagnostic character in *Tylos*, are shown for most of the lineages examined in Figure S1 (photographs for additional lineages of *T. punctatus* sensu lato can be found in [9]). We used a sample of *Helleria brevicornis* Ebner, 1868 as outgroup in a subset of the phylogenetic analyses. The monotypic *Helleria*, endemic to the northern Tyrrhenian area, is the only other genus of the family Tylidae. Genomic DNA was isolated from 2–4 legs per specimen with the DNeasy kit (Qiagen, Inc., Valencia, CA). We PCR-amplified segments of four mitochondrial genes: 16S rDNA; 12S rDNA; Cytochrome Oxidase Subunit I (COI); and Cytochrome b (Cytb); primer sequences and amplification conditions are provided in Table S2. PCR-amplified products were cleaned with Exonuclease and Shrimp Alkaline Phosphatase, and subsequently cycle sequenced at the University of Arizona Genetics Core. We used Sequencher 4.8 (Gene Codes, Ann Arbor, MI) for sequence editing and primer removal. None of the protein-coding sequences had premature stop codons or frame shifts, suggesting that they are not pseudogenes. All sequences were deposited in GenBank (Accession Numbers KJ468109–KJ468188).

### 2.2 Sequence Alignment

Non-protein-coding sequences were aligned with MAFFT v.6.0 [16], as implemented in http://mafft.cbrc.jp/alignment/server/(Accessed 2014 Feb 4), with the Q-INS-I strategy, which considers secondary structure of RNA, and with the L-INS-i strategy with default parameters (e.g. Gap Opening penalty = 1.53). Resulting alignments were edited manually within MacClade v.4.06 [17]. Regions for which homology could not be confidently established were identified with GBlocks v.0.91b [18], and excluded from the phylogenetic analyses. The following GBlocks parameters were used: “Allowed Gap Positions”  =  half; “Minimum Length Of A Block”  = 5 or 10; and “Maximum Number Of Contiguous Nonconserved Positions”  = 4 or 8. Alignments showing included and excluded positions are available in Datasets S1 & S2. Dataset S1 included *Helleria brevicornis* as the outgroup. High divergences between *H. brevicornis* and *Tylos*, however, rendered many positions in the two ribosomal genes unusable (Table 1). To increase the number of usable positions at the two ribosomal genes and reduce noise due to substitution saturation, we subsequently generated a dataset (Dataset S2) in which *H. brevicornis* was removed, and the above MAFFT and GBlocks procedures were repeated (see details about rooting of this dataset in the Results section).

**Table 1:** Description of characters and selected substitution models for Dataset S1.

Gene	Samples	Total characters^a^	Excluded characters^b^	Included characters	Parsimony informative	AICc (weight)	AIC (weight)	BIC (weight)
16S rDNA	73	498	324	174	79	K80+G (1.00)	HKY+I+G (0.26)	HKY+I+G (0.70)
12S rDNA	63	524	351	173	97	K80+G (0.99)	TIM2+G (0.23)	TrN+G (0.38)
Cytb	68	296	0	296	161	HKY+I+G (0.51)	TIM2+I+G(0.34)	TrN+I+G (0.35)
COI	67	600	0	600	233	HKY+I+G (0.36)	TPM2uf+I+G (0.23)	HKY+I+G (0.75)
MT	73^c^	1918	675	1243	570	TrN+I+G (0.30)	TrN+I+G (0.28)	HKY+I+G (0.77)

Number of characters per gene region that were excluded from and included in the phylogenetic analyses. The number of parsimony informative characters is based on included characters only. Best model selected by jModelTest according to each criterion (AIC, AICc, BIC) and its corresponding weight.

a Total number of characters in the alignment, including gaps.

b Criteria for character exclusion are described in a nexus file in the supporting information.

c The number of samples was selected for the combined analyses of mitochondrial genes, including missing genes of *Tylos* species.

MT  =  concatenated dataset of four mitochondrial genes.

### 2.3 Phylogenetic Analyses

Phylogenetic analyses were conducted with the sequences of the four loci concatenated into a single dataset. We used jModeltest v0.1.1 [19] to determine the most appropriate model of DNA substitution among 88 candidate models on a fixed BioNJ-JC tree, under the Akaike Information Criterion (AIC), corrected AIC(c), and Bayesian Information Criterion (BIC) (Tables 1 & 2). We used the closest more complex model (based on the BIC) available in the corresponding Maximum Likelihood (ML) and Bayesian analyses (see Tables S3 & 3), except that when a proportion of invariable sites (I) and a Gamma distribution of rates among sites (G) was selected according to jModeltest, we excluded parameter I to avoid problems related to dependency between both parameters (see RaxML manual and [20]). In addition, to assess robustness of the results to substitution model, we also used the complex model GTR+G. The following two data partitioning schemes were implemented: (a) all positions within a single partition; and (b) the best partitioning scheme according to the BIC implemented in PartitionFinder v.1.0 [21]. The following parameters were used in PartitionFinder: branch lengths  =  linked; models  =  mrbayes; model selection  =  BIC; search  =  greedy; and a priori partitioning by a combination of each gene and codon position.

**Table 2:** Description of characters and selected substitution models for Dataset S2 (excluding *H. brevicornis*).

Gene	Samples	Total characters^a^	Excluded characters^b^	Included characters	Parsimony informative	AIC (weight)	AICc (weight)	BIC (weight)
16S rDNA	72	499	144	355	216	TIM2+I+G (0.7004)	TIM2+I+G (0.3817)	TIM2+I+G (0.3914)
12S rDNA	62	525	201	324	197	TrN+G (0.2392)	TrN+G (0.7089)	TrN+G (0.7404)
Cytb	67	296	0	296	158	TIM2+I+G (0.2744)	TrN+I+G(0.4196)	TrN+I+G (0.4468)
COI	65	600	0	600	230	HKY+I+G (0.2949)	HKY+I+G (0.4869)	HKY+I+G (0.8347)
MT	72^c^	1920	345	1575	801	HKY+I+G (0.2983)	HKY+I+G (0.3601)	HKY+I+G (0.8917)

Number of characters per gene region that were excluded from and included in the phylogenetic analyses. The number of parsimony informative characters is based on included characters only. Best model selected by jModelTest according to each criterion (AIC, AICc, BIC) and its corresponding weight.

a Total number of characters in the alignment, including gaps.

b Criteria for character exclusion are described in a nexus file in the supporting information.

c The number of samples was selected for the combined analyses of mitochondrial genes, including missing genes of *Tylos* species.

MT  =  concatenated dataset of four mitochondrial genes.

For the ML analyses, three approaches were employed: (a) RaxML v.8.0.7 (“GTRGAMMA” model; standard bootstrap search) [22]; (b) GARLI v.2.0.1 [23] implemented in the CIPRES server [24], which uses genetic algorithms for the ML search; and (c) PhyML v.3.1 (search  =  SPR & NNI) [25]. Clade support within ML analyses was examined by: (a) the approximate Likelihood Ratio (aLRT) test using the Shimodaira-Hasegawa (SH-like) procedure, as implemented in PhyML; and (b) non-parametric bootstrap analyses (100–1000 replicates) in all three ML programs, and summarized with 50% majority rule consensus trees computed by the SumTrees script (v.3.3.1) implemented in DendroPy v.3.10.1 [26].

For the Bayesian analyses, two programs were used. The first one was MrBayes v.3.2.2 [27]–[29], but such analyses have been reported to return biased clade posterior probabilities in certain cases (e.g. the “star-tree paradox”; [30]–[32]). Therefore, we also applied two of the proposed strategies to alleviate such biases: the polytomy prior [33] as implemented in Phycas v.1.2.0 [34]; and a Gamma prior on the tree length as implemented in MrBayes v.3.2.2 [35]. The following criteria were used to evaluate convergence and adequate sampling of the posterior distribution: (a) Stable posterior probability values; (b) a high correlation between the split frequencies of independent runs as implemented in AWTY [36]; (c) small and stable average standard deviation of the split frequencies of independent runs; (d) Potential Scale Reduction Factor close to 1; and (e) an Effective Sample Size (ESS) >200 for the posterior probabilities and parameters, as evaluated in Tracer v.1.5 [37]. Tree samples prior to reaching a stationary posterior distribution were discarded (i.e., “burnin”), and the remaining samples were used to generate majority rule consensus trees with SumTrees (note: the tree summary function of Phycas was not used, as it returned incorrect clade posterior probabilities). Pairwise genetic distances with Kimura-2-parameter (K2P) correction were estimated with PAUPv.4.0b10 [38] for the four concatenated mitochondrial genes (Dataset S1) and for the COI gene separately; missing/ambiguous positions were removed for each pairwise sequence comparison.

## Results

**Figure 2:**
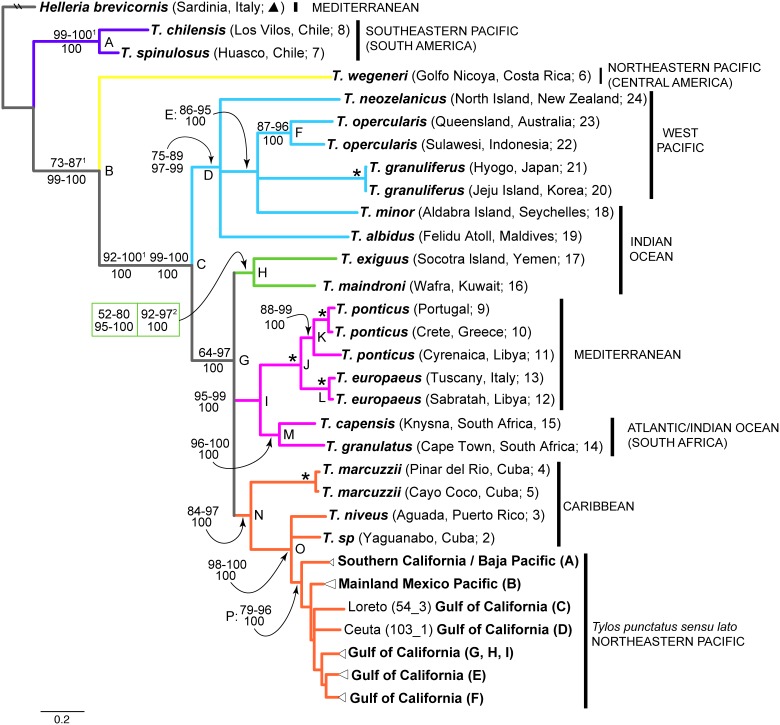
RaxML bootstrap majority rule consensus tree of the genus *Tylos*. Clades with<66% support were collapsed. Based on regular bootstrap partitioned analysis (based on PartitionFinder BIC) of Dataset S2 (excluding *Helleria brevicornis*, which was re-drawn manually). Clade colors correspond to clades in Fig. 1. Numbers by nodes indicate the corresponding range of Bootstrap Support and aLRT probabilities (BS; top) for Maximum likelihood (RaxML, Garli, and PhyML); and Posterior Probabilities (PP; bottom) for Bayesian inference methods (MrBayes and Phycas), including all partitioning schemes. * denotes nodes that received 100% support for all methods. ^1^Clade support values based on Datasets S1 analyses (see Table S3). ^2^Clade support values based on analyses of dataset excluding *H. brevicornis*, *T. chilensis*, *T. spinulosus*, and *T. wegeneri* (see text for details). All other support values are based on Dataset S2 (see Table 3).

The concatenated dataset of four mitochondrial genes (MT) including *H. brevicornis* (Dataset S1) retained 1243 characters (570 were parsimony informative), after removal of positions that could not be confidently aligned (Table 1). In contrast, Dataset S2 (excluding *H. brevicornis*) retained 1575 characters (801 were parsimony informative; Table 2). All Bayesian analyses achieved convergence and an adequate sample of the posterior distribution on the basis of the criteria outlined in the Methods section. Figure 2 depicts the inferred phylogenetic relationships among the *Tylos* species examined (the main lineages in the *T. punctatus* s. l clade are shown collapsed, as their relationships are addressed in detail by Hurtado et al. [9]), with ranges of clade support from the different methods and substitution models. Hereafter, Bootstrap proportions and aLRT probabilities are referred to as “BS”, whereas Posterior Probabilities are referred to as “PP” (clade support values for each method and substitution model are provided in Tables S3 & 3, respectively for Datasets S1 & S2). In general, the use of different substitution models or priors had little effect on clade support values, but some discrepancies were observed between ML and Bayesian analyses (Fig. 2 and Tables S3 and 3).

**Table 3:** Node support obtained from different methods and substitution models with the dataset that excluded *Helleria brevicornis* (Dataset S2).

	Analysis and partitioning scheme																	
	PhyML		RaxML		Garli			MrBayes									Phycas^5^	
Node	Part^1^: single HKY+G SH-like^2^	Part^1^: single HKY+G Boot^3^	Part^1^: single GTR+G Boot	Part: BIC GTR+G Boot	Part: single GTR+G Boot	Part: single HKY+G Boot	Part: BIC Boot	Part: single HKY+G BrLens^4^: 0.5	Part: single GTR+G BrLens: DF	Part: single GTR+G BrLens: 0.1	Part: single GTR+G BrLens: 0.5	Part: single GTR+G BrLens: 10	Part: BIC BrLens: DF	Part: BIC BrLens: 0.1	Part: BIC BrLens: 0.5	Part: BIC BrLens: 10	Part: single HKY+G	Part: single GTR+G
C	100	100	100	100	100	99	99	100	100	100	100	100	100	100	100	100	100	100
D	89	80	82	75	79	88	75	99	98	98	99	99	97	97	97	97	98	97
E	92	95	86	87	88	88	87	100	100	100	100	100	100	100	100	100	100	100
F	96	91	91	87	91	88	90	100	100	100	100	100	100	100	100	100	100	100
G	97	81	76	64	73	67	69	100	100	100	100	100	99	100	100	100	100	100
H	80	70	59	52	61	52	63	97	97	97	97	96	99	99	99	100	97	95
I	98	99	99	97	95	99	97	100	100	100	100	100	100	100	100	100	100	100
J	100	100	100	100	100	100	100	100	100	100	100	100	100	100	100	100	100	100
K	95	95	97	96	94	97	88	100	100	100	100	100	100	100	100	100	100	100
L	100	100	100	100	100	100	100	100	100	100	100	100	100	100	100	100	100	100
M	96	100	100	99	99	100	96	100	100	100	100	100	100	100	100	100	100	100
N	97	92	96	88	84	94	84	100	100	100	100	100	100	100	100	100	100	100
O	100	100	100	100	100	99	98	100	100	100	100	100	100	100	100	100	100	100
P	96	86	82	82	79	88	86	100	100	100	100	100	100	100	100	100	100	100

Tree was rooted at node C (i.e., ougroup  =  *T. spinulosus*, *T. chilensis*, and *T. wegeneri*). Percent bootstrap support for Maximum Likelihood (PhyML, RaxML and Garli), SH-like-aLRT probability (PhyML only), and percent posterior probability for Bayesian analyses (MrBayes and Phycas).

1 Part.  =  Partitioning Scheme (single  =  one partition; BIC  =  Best partitioning scheme according to PartitionFinder Bayesian Information Criterion). BIC = 12S rDNA+16S rDNA (GTR+G); Cytb codon 1 (K80+G); Cytb codon 2+ COI codon 2 (HKY+G); Cytb codon 3+ COI codon 3 (HKY+G); COI codon 1 (SYM + G).

2 Boot  =  Bootstrap search.

3 SH-like  =  approximate Likelihood Ratio Test (aLRT) for branch lengths with the Shimodaria-Hasegawa-like procedure.

4 BrLens  =  Branch length Priors used in MrBayes v.3.2.2. DF  =  default. 0.1; 0.5; and 10 refer to the prior value assigned to *β_T_* under the Gamma prior on tree length (*α_T_*, *α*, and *c,* were assigned a value of 1 in all cases; e.g. command “prset brlenspr = unconstrained:gammadir (1,0.1,1,1)”, where gammadir (*α_T_*,*β_T_*,*α*,*c*).

5 A polytomy prior was assumed in Phycas analyses.

A most basal split within the genus is observed between a clade (clade A: 99–100 BS; 100 PP; purple in Figs. 1 & 2) that contains the two species from Chile (i.e., *T. spinulosus* Dana, 1853 and *T. chilensis* Schultz**,** 1983), and a clade (B; Fig. 2) comprised of the remaining species (73–87 BS; 99–100 PP; based on Datasets S1). Kimura-2-parameter divergences between clade A and clade B ranged between 21 and 43% for the four genes combined, and between 20 and 32% for the COI gene alone (Tables S4 & S5, respectively). Within clade B, a most basal divergence was observed between *T. wegeneri* Vandel, 1952 (sample from the Pacific coast of Costa Rica; yellow), and a clade (C: 92–100 BS and 100 PP with Datasets S1; 99–100 BS and 100 PP with Dataset S2) that contained the remaining lineages. Hereafter, unless otherwise noted, clade support is described only for Dataset S2, which assumed the monophyly of clade C (i.e., *T. chilensis*, *T. spinulosus*, and *T. wegeneri* were used as the outgroup). Clade C was comprised of two main lineages: clade D (blue; 75–89 BS; 97–99 PP) distributed in the West Pacific and Indian Ocean; and clade G (64–97 BS; 100 PP). Within clade D, a basal polytomy of three lineages was observed: *T. albidus* Budde-Lund, 1885 (Indian Ocean); *T. neozelanicus* Chilton, 1901 (West Pacific); and clade E (86–95 BS; 100 PP; West Pacific), which was in turn comprised of *T. granuliferus* Budde-Lund, 1885, *T. opercularis* Budde-Lund, 1885 (clade F; one lineage from Australia and one from Indonesia), and *T. minor* Dollfus, 1893.

Clade G was comprised of three main clades (H, I, and N) joined at a basal polytomy. Clade H (green), which is comprised of two species from the Arabian Peninsula (*T. exiguus* Stebbing, 1910 from Socotra Island, Indian Ocean, and *T. maindroni* Giordani Soika, 1954 from Kuwait, Persian Gulf), received<50% support with Datasets S1 (not shown), variable support with Dataset S2 (52–80 BS; 95–100 PP), and higher support (92–97 BS; 100 PP) with a third dataset, which was identical to Dataset S2 except that the three most basal species of *Tylos* (i.e., *T. chilensis*, *T. spinulosus*, and *T. wegeneri*) were excluded, and rooting was performed at the branch joining clades D and G. This last dataset did not result in an increased number of usable positions, but likely reduced noise, stemming from the high divergence of *T. chilensis*, *T. spinulosus*, and *T. wegeneri*. Clade I (magenta; 95–99 BS; 100 PP) was comprised of a highly supported (100 BS and PP) Mediterranean Sea lineage (clade J; made up of *T. ponticus* Grebnitzky, 1874 and *T. europaeus* Arcangeli, 1938) and of a South African clade (M: 96–100 BS; 100 PP; made up of *T. capensis* Krauss, 1843 and *T. granulatus* Krauss, 1843). Clade N (orange; 84–97 BS; 100 PP) was comprised of lineages from the Caribbean (i.e., *T. marcuzzi* Giordani Soika, 1954**,** *T. niveus* Budde-Lund, 1885, and *T. sp.* from Yaguanabo, Cuba), and the southern California/Pacific Mexico region. Within clade N, *T. marcuzzii* was sister to the remaining lineages (clade O: 98–100 BS and 100 PP). Within clade O, *T. punctatus* sensu lato formed a monophyletic group (clade P: 79–96 BS; 100 PP), whose closest relatives, *T. niveus* and/or *T. sp.* (Yaguanabo), are distributed in the Caribbean.

## Discussion

Our results provide insights into the evolutionary history of the genus *Tylos*, a lineage that successfully colonized and diversified within the harsh sea-land interface at a global scale. Although we acknowledge that phylogenetic inferences based on multiple unlinked markers are desirable (reviewed in [39]), we were unable to obtain nuclear gene sequences despite numerous attempts, likely due to DNA degradation in relatively old samples. Nonetheless, given that phylogenetic relationships among species of *Tylos* have not been studied before, the inferred mitochondrial phylogeny currently represents the most plausible phylogenetic hypothesis for this genus. Our results reveal the presence of highly divergent clades that, in general, group according to geography (Figs. 1 & 2). Relationships among the major clades and their approximate distributions based on morphological records are shown depicted with colors in Fig. 1. Below we discuss phylogenetic and distributional patterns of *Tylos*, which provide important clues on the biogeography and evolution of this group.

The two species from Chile, *T. chilensis* and *T. spinulosus*, formed a well-supported monophyletic group (clade A). Divergence of this lineage represents the most basal split within *Tylos*, suggesting a long history of presence and isolation in the southern East Pacific. The divergence of the *T. wegeneri* lineage, which is also found in the East Pacific region, represents the second most basal split, also implying a long history of isolation. This species, however, was originally described from Venezuela [40], and has also been reported in Tobago and Saint Martin, in the Caribbean, and in the Atlantic coast of Florida [11]; whereas in the Pacific, it has only been reported in Costa Rica. Caribbean-Atlantic specimens assigned to *T. wegeneri* must be examined to establish whether they are closely related to our *T. wegeneri* sample. If so, the Pacific/Caribbean-Atlantic distribution of this lineage must have been achieved prior to the closure of the Panama Isthmus. Within the supralittoral isopod genus *Ligia*, a genetically distinct and diverse clade (representing what a appears to be complex of cryptic species currently assigned to *Ligia baudiniana* Milne-Edwards, 1840), also occupies both, the Caribbean/Atlantic and Pacific coasts of this region, but lineages from the Pacific are highly divergent from those in the Caribbean/Atlantic [41]. A similar pattern is possible for *T. wegeneri*.

The remaining species of *Tylos* examined are grouped within clade C. Lineages D, H, I and N, which make up clade C, are highly divergent from each other (15–41% at the four genes combined; Table S4), and occupy broad and distinct geographic regions. Clade D, which appears to be the most basal of these lineages, has a West Pacific/Indian Ocean distribution and high within-clade divergences (up to 36% at the four genes; Table S4). In the West Pacific, *T. opercularis* is reported from the Philippines, Papua New Guinea, Sulawesi, and the eastern coast of Australia. *Tylos granuliferus* is reported in Japan, Korea and Eastern Russia (Vladivostok and southernmost Kuril Islands), whereas *T. neozelanicus* is reported in New Zealand. In the Indian Ocean, *T. minor* is reported in east Africa along the coasts of southern Somalia and Kenya, as well as in the islands of Madagascar, Seychelles, Aldabra, and Comoro; whereas *T. albidus* is reported in islands farther east (i.e., Nicobar, Maldives, and Sri Lanka) [11]. Given the geographic distribution of clade D, it is likely that *T. australis* Lewis & Bishop, 1990 (from southeastern Australia), *T. nudulus* Budde-Lund, 1906 (from Christmas Island, south of Java), and *T. tantabiddyi* Lewis, 1991 (from Western Australia), which were not included in this study, belong to this clade.

Our analyses failed to resolve the relationships among clades H, I, and N, resulting in a basal polytomy within clade G. Clade H, which was supported by a subset of the analyses, contained *T. exiguus* and *T. maindroni*, two species that are highly divergent from each other (22% at COI; Table S5). Both are distributed in the Arabian Peninsula, the largest peninsula in the world, but on opposite sides: *T. exiguus* is reported in the Red Sea coasts and Socotra Island (locality 17; Fig. 1); whereas *T. maindrioni* is reported in the Persian Gulf, on the eastern side of the peninsula [11]. The earliest date at which the *T. exiguus* lineage could have colonized the Red Sea and Gulf of Aden region is ∼25 Ma; when these basins are hypothesized to have formed [42]. Clade I, the second lineage within clade G, is distributed in: (a) southern Africa (*T. granulatus* is reported from northern Namibia to Cape Town, South Africa; whereas *T. capensis* is reported from Cape Town to Port Elizabeth, South Africa); (b) northwestern Africa as far south as Dakar, Senegal (*T. ponticus*) [11], (c) the Atlantic Ocean islands of Azores (*T. europaeus*), Madeira (*T. ponticus*), and the Canary Islands (*T. ponticus*); (d) the Atlantic coast of Europe, as far north as Bretagne, France (*T. europaeus*); and (e) the Mediterranean and Black seas (*T. europaeus* and *T. ponticus*). Given the above patterns, it is likely that *T. madeirae* Arcangeli, 1938 (from Madeira Island; [11]), not included in our study, belongs to clade I. Clade N, the third lineage within clade G, is reported from Florida, Bahamas, and the Caribbean (*T. marcuzzii* and *T. niveus*), as well as from Bermuda (*T. niveus*), Brazil (*T. niveus*), and the northeastern Pacific (*T. punctatus* s.l.), in the region between southern California and Central Mexico, including the Gulf of California [9], [11].

The phylogeographic patterns of members of *Tylos* could have been shaped by several processes: break-up of land masses leading to vicariance; overwater dispersal (particularly over relatively short distances, given the biology of this isopod); range expansions and contractions associated with eustatic sea level and climate changes; and ecological speciation and/or niche partitioning. Unfortunately, our interpretation of evolutionary patterns is somewhat constrained by the lack of reliable molecular clock calibration points (fossils or vicariant events), or substitution rates that would enable inference of meaningful estimates of divergence times among *Tylos* lineages. Although large divergences among the most basal clades suggest ancient splits, their timing is uncertain. Several lines of evidence are consistent with an ancient origin for the genus *Tylos*. Tylidae (and Ligiidae) occupy the most basal positions within Oniscidea [4], [5], a suborder whose origin is estimated around the Paleozoic (∼300 Ma) [43]. Although fossils of *Tylos* have not been recovered, fossils assigned to *Ligia* are dated at ∼110 Ma, whereas the earliest oniscidean fossils exhibiting morphological characteristics of extant woodlice (which are more derived than Tylidae and Ligiidae), are dated at ∼30 Ma, in the late Eocene [43]. In addition, the cosmopolitan distribution of *Tylos* has been regarded as an indication of an ancient origin [44]. The biology of *Tylos* (i.e., lack of a planktonic larval stage, extremely limited swimming abilities, and inability to survive under water beyond a few hours) may have prevented dispersal across vast oceanic scales. Therefore, we consider that tectonic events such as the breakup of Gondwana, rather than trans-oceanic dispersal, explain the presence of *Tylos* on different continents. Although the sequence and timing of events are controversial, the breakup of Gondwana is dated at ∼160–80 Ma (million years ago) [45]. These events could have led to the divergence of clades D (Indian Ocean/West Pacific), I (Eastern Atlantic: Mediterranean and Africa coasts), and N (Caribbean and Northeastern Pacific). Accordingly, the split that led to clade A (*T. chilensis + T. spinulosus*), as well as the split that led to *T. wegeneri*, may predate the breakup of Gondwana.

Overwater dispersal, however, likely played a role in the colonization of certain localities of the West Pacific/Indian Ocean, facilitated by the geographic proximity among some islands and between some mainland and island localities. Similarly, overwater dispersal may have occurred in the eastern Atlantic (see below), between the Caribbean and the northeastern Pacific, and within the Caribbean (although proto-Antillean vicariance [46] or a hypothesized temporary land bridge ∼33–35 Ma [47], represent alternative hypotheses for colonization of Caribbean islands). Recent overwater dispersal probably explains the presence of *T. niveus* in Bermuda, a highly isolated volcanic island north of the Caribbean, which appears to have been submerged at several instances during the Pleistocene [48], [49]. In addition to *Tylos*, a member of *Ligia* is also reported in Bermuda; both of these semiterrestrial taxa likely arrived to this island via rafting [50]. The phylogenetic affinities of *Tylos* populations found in Bermuda, however, need to be examined to identify their possible origin.

The timing of the divergence between the southern Africa and Mediterranean clades is also uncertain (19.7–24.7% divergence at COI; Table S5). Colonization of the Mediterranean, however, probably occurred after the Messinian Salinity Crisis (∼6–5.3 Ma), a period during which the Mediterranean basin was either completely dry or hypersaline [51]; conditions that would likely have precluded the presence of *Tylos*. It is unclear whether the split between *T. ponticus* and *T. europaeus* (17–20% at COI; Table S5) occurred before or after the Messinian Salinity Crisis. These two species have different ecologies, which may enable their coexistence in the same regions, but not in the same microhabitat: *T. europaeus* occurs in fine grain sandy beaches, whereas *T. ponticus* inhabits coarse sand or pebble beaches [44]. It is therefore possible that their divergence was associated with ecological speciation. *Tylos europaeus* appears to be competitively excluded from very coarse-grained beaches, whereas *T. ponticus* can tolerate a broader range of sediment grain sizes [52]. Overwater dispersal likely explains the distribution of these species in the volcanic islands of Azores and Madeira, but the phylogenetic affinities of these populations have not been determined.

*Tylos* lineages found in the Pacific coast between southern California and Central Mexico, including the Gulf of California, have a close relationship with lineages from the Caribbean. The closest relatives of these northeastern Pacific lineages were *T. niveus* and a sample from Yanaguabo, Cuba. The latter could not be identified to species on the basis of morphology because the specimen was severely damaged, but given its high divergence from *T. niveus* (16% at COI; Table S5), it probably represents an undescribed species. The ancestor of the southern California-Pacific Mexico-*T. niveus*-‘Yaguanabo sample’ clade (clade O) was likely distributed in the Caribbean, because *T. marcuzzii*, its sister lineage, has a Caribbean distribution. Therefore, colonization of the northeastern Pacific appears to have proceeded from the Caribbean, which is congruent with paleontological studies of the Gulf of California reporting that most fauna-rich sediments found in this region have affinities with Caribbean fauna [53]. Marine fossils with Caribbean affinities in the Gulf of California date back to Miocene times [54]. Interestingly, Gulf of California/Caribbean affinities are also evident in the phylogeographic patterns of intertidal isopods of the genus *Excirolana* [55]. As discussed above, the only other *Tylos* species reported from the Caribbean, *T. wegeneri*, was found to be very divergent from the northeastern Pacific or other Caribbean lineages (28–36%; Table S4).

Comparatively, the history of *Tylos* in the northeastern Pacific region from southern California to central Mexico is much more recent than in the southern East Pacific. Nonetheless, high levels of cryptic diversity are observed in this region. Phylogeographic patterns indicate the presence of highly divergent lineages (up to 20% K2P COI divergences) [9], in a region where only one currently accepted species (i.e., *T. punctatus*) is reported. Based on these patterns, however, Hurtado et al. [9] indicate that *T. punctatus* sensu stricto is restricted to the Pacific coast region between southern California and Central Baja California, and that the divergent lineages found within the Gulf of California and south of this basin, in Central Pacific Mexico, appear to correspond to a complex of cryptic species (*T. punctatus* s. l.), which form a monophyletic group with *T. punctatus*. Phylogeographic patterns of these lineages are discussed in detail in Hurtado et al. [9]. The complex geological history of the Gulf of California, a basin that is suggested to have formed at least ∼12Ma [56], likely played a role in the extraordinary diversification of the supralittoral *Tylos* and *Ligia* within this basin [9], [10].

Supralittoral isopods were suggested to be highly dispersive species [44]. Recent phylogeographic studies, however, challenge this early proposal for *Tylos* and *Ligia*, in which high levels of genetic differentiation and cryptic diversity are observed at small geographic scales [8]–[10], [41], [57], [58]. This is consistent with both, the biological characteristics that confer these isopods low vagility, and the fragmented nature of their habitats. High levels of genetic differentiation and cryptic diversity appear to be common for *Tylos* in different regions of the world. High levels of allopatric cryptic diversity and isolation occurred in the northeastern Pacific [9]. High genetic divergence is observed between the samples from Puerto Rico and Yaguanabo, Cuba (14.1 and 16% K2P; respectively at the four gene and COI datasets; Tables S4 & S5), and further cryptic diversity may be found at other Caribbean locations. The two samples of *T. opercularis* (from Sulawesi and Australia) were highly divergent (14.4%; Table S4), possibly representing different species. Large intra-specific genetic divergences were also observed in *T. ponticus* from Libya vs. Greece and Portugal (up to 14.8%; Table S4); whereas divergence between *T. europaeus* from Libya and Italy was 5% (Table S4). Divergence between the two *T. marcuzzii* localities collected within Cuba is 2.5% (Table S4). Low genetic divergence, however, was observed between *T. granuliferus* from Japan and Korea (0.3%; Table S4). Similarly, low genetic divergence (≤0.6% COI) is reported in *T. punctatus* s. s. (as defined by [9]), probably reflecting a drastic bottleneck and a recent postglacial expansion in its current range in the Pacific region between southern California and central Baja California [9].

Taxonomic confusion has probably contributed to overestimation of the dispersal potential of *Tylos*. Specimens from many localities around the world were incorrectly assigned to *T. latreillii* Audouin 1826 (e.g. from the Mediterranean, East Africa, Atlantic coast of Europe, Caribbean, Bermuda, and the Gulf of California [15], [44], [59]–[61]), leading to a misconception of high dispersal potential [15], [44]. Originally described from an unspecified location in Egypt [13], *T. latreillii* currently lacks type specimens and a good description, rendering it a *nomen dubium* [14]. It probably corresponds to *T. europaeus* or *T. ponticus* from the Mediterranean Sea [14], or to *T. exiguus* from the Red Sea [62]. Nonetheless, extensive sampling reveals that none of the above three species are present in the Gulf of California [9]. Similarly, these three species are unlikely to inhabit the Caribbean region or Bermuda, where occurrence reports of specimens with affinity to “*T. latreilli*” await verification [11]. Another case that may have contributed to an overestimation of the dispersal potential of *Tylos* is that of *Tylos insularis* Van Name, 1936, from the Galapagos Islands. Although currently considered a synonymy of *T. punctatus* [11], the morphology of the ventral plates of the fifth pleonite of Galapagos samples [63] is very different to that of *T. punctatus* s. s. [60], or any of the cryptic lineages found in the Gulf of California and central Pacific Mexico [9]. This, in addition to their geographic separation, indicates that *T. insularis* likely represents a distinct species, as proposed by Van Name [60].

## Conclusion

The inferred mitochondrial phylogeny of 17 of the 21 currently recognized *Tylos* species sheds light on the phylogenetic relationships within this globally widespread supralittoral-endemic genus. Our results reveal the presence of highly divergent clades within *Tylos* that have relatively discrete, yet broad, distributions. The most basal divergences involve lineages distributed in the southern East Pacific, implying a long history of isolation in this region. The remaining lineages are grouped in a clade, in which the most basal divergence involves a lineage made up of West Pacific and Indian Ocean taxa. Sister to this lineage, is a clade that has three deeply divergent lineages: one with taxa from the Arabian Peninsula; a second one with the taxa from the Mediterranean Sea and South Africa; and a third one with taxa from the Caribbean and the northeastern Pacific. Colonization of the northeastern Pacific appears to have proceeded from the Caribbean.

Divergences of lineages from different continents (e.g. clades D, I, and N) were probably shaped by tectonics. Although biological characteristics of *Tylos* may have prevented dispersal of this isopod across vast oceanic scales, overwater dispersal likely enabled range expansions within some basins, and colonization of volcanic islands. In addition, present-day distributions were likely influenced by changes in sea level, which can alter habitat availability and connectivity, as well as changes in climate, which can cause range contraction and expansion, particularly at the latitudinal limits of this tropical/subtropical taxon (e.g. in the southern California-northern Baja California Peninsula region [9]).

Our findings imply that the dispersal abilities of *Tylos* are more limited than previously thought. High levels of cryptic genetic diversity are observed in different regions of the world. Therefore, a taxonomic revision of this group is necessary. Furthermore, new collections would enable examination of nuclear markers to corroborate our findings. Finally, more detailed sampling and ecological characterization within regions may reveal additional divergent lineages, and provide better insight into phylogeographic patterns and the mechanisms of diversification of this widespread yet poorly studied taxon.

## Supporting Information

Figure S1 Ventral shape of the fifth pleonite for representative samples from 17 species. (13 from this study; 4 from previous studies).
(PDF)

Table S1 Information on *Tylos* specimens used in the phylogenetic analyses.
(DOCX)

Table S2 PCR primers information and annealing temperature (Tm).
(DOCX)

Table S3 Node support obtained from different methods and substitution models for analyses of Datasets S1.
(DOCX)

Table S4 Percent Kimura-2-parameter distances for the concatenated dataset of four mitochondrial genes (MT; 1243 characters).
(DOC)

Table S5 Percent Kimura-2-parameter distances for the COI gene (600 characters).
(DOC)

Dataset S1 Nexus file containing the alignment of all the taxa used in the analyses. Alignments are annotated by gene, and positions that were excluded and included (“charset trust_always”) in the phylogenetic analyses are identified.
(NEX)

Dataset S2 Nexus file containing the alignment of all the taxa used in the analyses with the exception of *Helleria brevicornis.* Alignments are annotated by gene, and positions that were excluded and included (“charset trust_always”) in the phylogenetic analyses are identified.
(NEX)
