# Supplementary material for: Global Diversification at the Harsh Sea-Land Interface: Mitochondrial Phylogeny of the Supralittoral Isopod Genus Tylos (Tylidae, Oniscidea)
Source: PLoS One. 2014 Apr 15;9(4):e94081. doi: 10.1371/journal.pone.0094081 (PMC3988090; doi:10.1371/journal.pone.0094081)
Supplement: Figure S1 — Ventral shape of the fifth pleonite for representative samples from 17 species. (13 from this study; 4 from previous studies). (PDF) [file pone.0094081.s001.pdf]

Figure S1. Ventral shape of the fifth pleonite for representative samples from 17 species (13 from this study; 4 from previous studies).

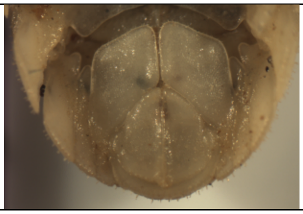	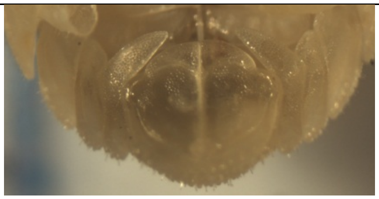	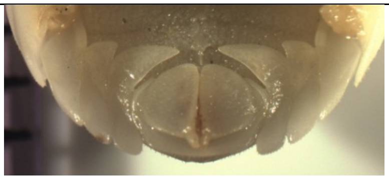	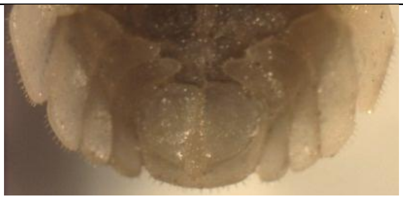	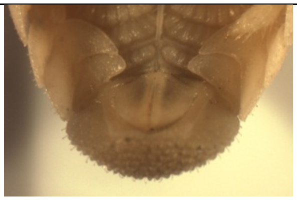
**1. *Tylos niveus***<br>28_1, Puerto Rico (Aguada)	**2.*Tylos marcuzzii***<br>Tmar_2, Cuba (Pinar del Rio)	**3.*Tylos spinulosus***<br>Cal_1, Chile (Caleta Punta Choros)	**4.*Tylos chilensis***<br>CH_1, Chile (Punta Tablas)	**5.*Tylos wegeneri***<br>#08, Costa Rica (Golfo Nicoya)
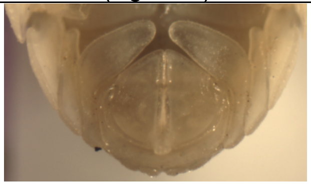	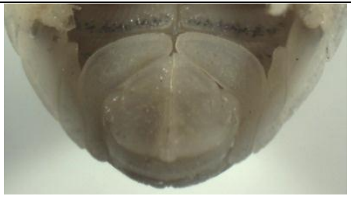	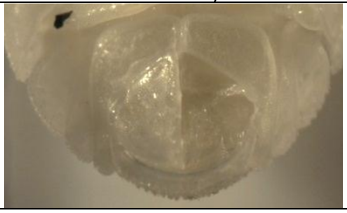	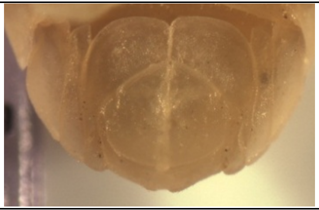	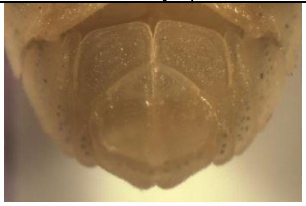
**6-1.*Tylos granuliferus***<br>Hyo_2, Japan (Hyogo)	**6-2.*Tylos granuliferus***<br>CY_1, Korea (Jeju Island)	**7.*Tylos albidus***<br>Ta_1, Maldives Islands	**8-1.*Tylos opercularis***<br>To_1, Sulawesi (Palu)	**8-2.*Tylos opercularis***<br>To_2, Australia (Queensland)
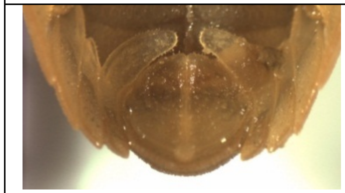	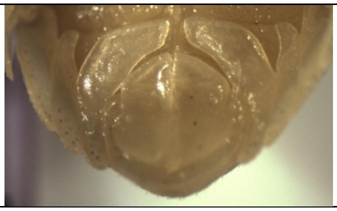	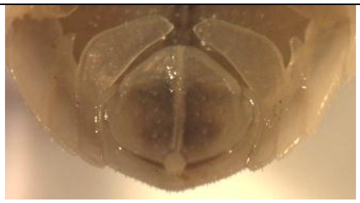	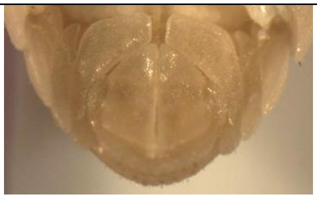	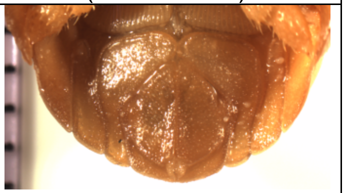
**9. *Tylos europaeus***<br>25_2, Italy (Tuscany)	**10. *Tylos ponticus***<br>27_2, Greece (Crete)	**11. *Tylos maindroni***<br>Tma_1, Kuwait (Wafra)	**12. *Tylos exiguus***<br>Tex_1, Yemen (Socotra Island)	**13. *Tylos granulatus***<br>Gr_1, South Africa (Cape town)
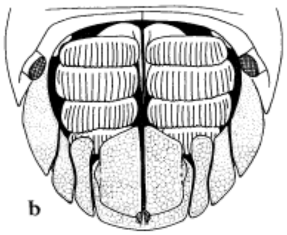	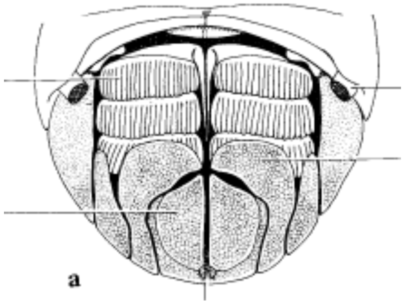	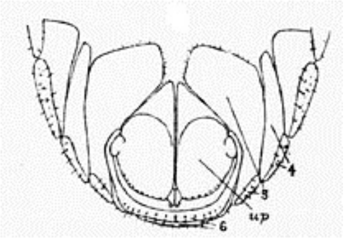	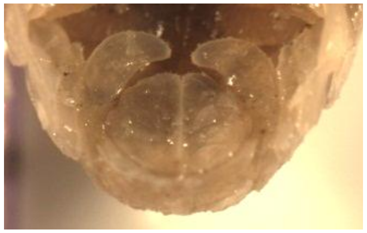	
**14*. Tylos capensis*** (from Kensley 1974) [1]	**15. *Tylos granulatus*** (from Kensley 1974) [1]	**16. *Tylos insularis*** (from Van Name 1924) [2]	***17. Tylos punctatus***<br>San Diego, California<br>**USNM 89583 (syntype)** (from [3])	

References

1. Kensley B (1974) Aspects of the biology and ecology of the genus *Tylos* Latreille. Ann S Afr Mus 65: 401-471.
2. Van Name WG (1924) Isopods from the Williams Galapagos Expedition. Zoologica 5: 181–210.
3. Hurtado LA, Lee EJ, Mateos M (2013) Contrasting phylogeography of sandy vs. rocky supralittoral isopods in the megadiverse and geologically dynamic Gulf of California and adjacent areas. PLoS ONE 8: e67827. doi: 10.1371/journal.pone.0067827.
