# Supplementary material for: Global Diversification at the Harsh Sea-Land Interface: Mitochondrial Phylogeny of the Supralittoral Isopod Genus Tylos (Tylidae, Oniscidea)
Source: PLoS One. 2014 Apr 15;9(4):e94081. doi: 10.1371/journal.pone.0094081 (PMC3988090; doi:10.1371/journal.pone.0094081)
Supplement: Table S1 — Information on Tylos specimens used in the phylogenetic analyses. (DOCX) [file pone.0094081.s002.docx]

**Table S1. Information on *Tylos* specimens used in the phylogenetic analyses.**

Sample numbers correspond to numbers in Figures 1 and 2.

| Number | Species | Locality | Country | Year | Sequence  ID | GenBank Accession Nos. (16S rDNA, 12S rDNA, Cytb, COI) | Voucher ID |
| --- | --- | --- | --- | --- | --- | --- | --- |
| 1 | *T. punctatus*^1^ |  |  |  |  | Multiple published sequences^1^ |  |
| 2 | *T. sp*^1^ | Yaguanabo, Cienfuegos | Cuba | 2010 | CU | KF007549, KF007888, KF007770, KF007724 |  |
| 3 | *T. niveus*^3^ | Aguada | Puerto Rico | 2011 | 28 | KJ468181, KJ468160, KF007771, KJ468120 |  |
| 4 | *T. marcuzzii*^2^ | Maria La Gorda, Pinar del Rio | Cuba | 2002 | Tmar2 | KJ468178, KJ468158, KJ468138, KJ468118 | MZUF 8660 |
| 5 | *T. marcuzzii*^2^ | Ciego de Avila, Cayo Coco | Cuba | 2002 | Tmar1 | KJ468177, KJ468157, n/a, KJ468117 | MZUF 8659 |
| 6 | *T. wegeneri*^4^ | Golfo Nicoya | Costa Rica | 1985 | #08 | KJ468188, KJ468165, KJ468146, KJ468126 | LACM 25 July 1985 J.A.Vargas |
| 7 | *T. spinulosus*^2^ | Atacama | Chile | 1980 | Ts1 | KJ468187, KJ468164, KJ468145, KJ468125 | MZUF 1096 |
| 8 | *T. chilensis*^2^ | Los Vilos, Punta Tablas | Chile | 1980 | Tch1 | KJ468168, KJ468148, KJ468129, KJ468109 | MZUF 279 |
| 9 | *T. ponticus*^5^ | Unknown | Portugal | 2009 | 428-1 | KJ468185, KJ468162, KJ468143, KJ468123 |  |
| 10 | *T. ponticus*^2^ | Preveli Beach, Crete | Greece |  | 27 | KJ468184, KJ468161, KJ468142, KJ468122 | MZUF 8398 |
| 11 | *T. ponticus*^2^ | Susah, Cyrenaica | Libya | 2008 | Tp1 | KJ468186, KJ468163, KJ468144, KJ468124 | MZUF 9447 |
| 12 | *T. europaeus*^2^ | Sabratah | Libya | 2005 | Te1 | KJ468170, KJ468150, KJ468131, KJ468111 | MZUF 9445 |
| 13 | *T. europaeus*^2^ | Burano, Tuscany | Italy |  | 25 | KJ468169, KJ468149, KJ468130, KJ468110 | MZUF 2295 |
| 14 | *T. granulatus*^2^ | Cape Town, Rondeberg | South Africa | 1980 | Tg1 | KJ468172, KJ468152, KJ468133, n/a | MZUF 219 |
| 15 | *T. capensis*^2^ | Knysna | South Africa | 1980 | Tc1 | KJ468167, KJ468147, KJ468128, n/a | MZUF 220 |
| 16 | *T. maindroni*^2^ | Wafra | Kuwait | 1988 | Tma1 | KJ468176, KJ468156, KJ468137, KJ468116 | MZUF 2156 |
| 17 | *T. exiguus*^2^ | Qalansiyah, Socotra Island | Yemen | 2000 | Tex1 | KJ468171, KJ468151, KJ468132, KJ468112 | MZUF 8687 |
| 18 | *T. minor*^2^ | Aldabra Island, Grande Terre | Seychelles | 1975 | Tm1 | KJ468179, n/a, n/a, n/a | MZUF 1249 |
| 19 | *T. albidus*^2^ | Felidu Atoll | Maldives | 1994 | Ta1 | KJ468166, n/a, KJ468127, n/a | MZUF 9104 |
| 20 | *T. granuliferus*^6^ | AnDeok, Jeju island | South Korea | 1993 | CY | KJ468173, KJ468153, KJ468134, KJ468113 |  |
| 21 | *T. granuliferus*^7^ | Toyooka, Hyogo | Japan | 2011 | HYO | KJ468174, KJ468154, KJ468135, KJ468114 |  |
| 22 | *T. opercularis*^2^ | Palu, Sulawesi | Indonesia | 1980 | To1 | KJ468182, n/a, KJ468140, n/a | MZUF 3244 |
| 23 | *T. opercularis*^2^ | Cape Tribulation, Queensland | Australia | 2004 | To2 | KJ468183, n/a, KJ468141, KJ468121 | MZUF 9425 |
| 24 | *T. neozelanicus*^2^ | Piha Beach, North Island | New Zealand | 2004 | Tnz1 | KJ468180, KJ468159, KJ468139, KJ468119 | MZUF 8869 |
|  | *H.* *brevicornis*^2^ | Burcei, Sardinia | Italy | 2011 | Hb | KJ468175, KJ468155, KJ468136, KJ468115 | MZUF 9448 |

^1^ Sequences from Hurtado et al. (2013)

^2^ Museo di Storia Naturale "La Specola", Zoological section, in Florence, Italy

^3^ Dr. Luis Hurtado (Texas A&M University, U.S.A)

^4^ Natural History Museum of Los Angeles County

^5^ Dr. Jonathan Wright (Pomona College)

^6^ Dr. Do Heon Kwon (Inje University, South Korea)

^7^ Miyuki Niikura (University of Tsukuba, Japan)
