# Supplementary material for: Global Diversification at the Harsh Sea-Land Interface: Mitochondrial Phylogeny of the Supralittoral Isopod Genus Tylos (Tylidae, Oniscidea)
Source: PLoS One. 2014 Apr 15;9(4):e94081. doi: 10.1371/journal.pone.0094081 (PMC3988090; doi:10.1371/journal.pone.0094081)
Supplement: Table S3 — Node support obtained from different methods and substitution models for analyses of Datasets S1. (DOCX) [file pone.0094081.s004.docx]

**Table S3. Node support obtained from different methods and substitution models for dataset including *Helleria brevicornis* (Dataset S1).**

Percent bootstrap support for Maximum Likelihood (PhyML, RaxML and Garli), SH-like-aLRT probability (PhyML only), and percent posterior probability for Bayesian analyses (MrBayes and Phycas). Nodes are shown in Fig. 2. Nodes F, G, and H had < 53% support, but exhibited higher support values in the analyses excluding *H. brevicornis* (see Fig. 2 and Table S4).

|  | Analysis and partitioning scheme | | | | | | | | | | | | | | |
| --- | --- | --- | --- | --- | --- | --- | --- | --- | --- | --- | --- | --- | --- | --- | --- |
|  | PhyML | | RaxML | | Garli | | | MrBayes | | | | | | Phycas^5^ | |
| Node | Part^1^: single HKY+G SH-like^2^ | Part: single HKY+G Boot^3^ | Part: single GTR+G Boot | Part: BIC GTR+G Boot | Part: single HKY+G Boot | Part: single GTR+G Boot | Part: BIC Boot | Part: single HKY+G BrLens^4^:0.5 | Part: single GTR+G; BrLens:DF | Part: single GTR+G BrLens:0.5 | Part: BIC BrLens:DF | Part: BIC BrLens:0.5 | Part: BIC BrLens:10 | Part: single HKY+G | Part: single GTR+G |
| A | 99 | 100 | 100 | 99 | 99 | 99 | 100 | 100 | 100 | 100 | 100 | 100 | 100 | 100 | 100 |
| B | 81 | 82 | 79 | 76 | 87 | 73 | 86 | 99 | 99 | 99 | 99 | 99 | 99 | 100 | 100 |
| C | 100 | 94 | 94 | 97 | 92 | 97 | 97 | 100 | 100 | 100 | 100 | 100 | 100 | 100 | 100 |
| D | 95 | 81 | 81 | 81 | 69 | 73 | 87 | 99 | 100 | 100 | 100 | 100 | 100 | 99 | 100 |
| E | 84 | 54 | 56 | 60 | <50 | <50 | 53 | 98 | 98 | 99 | 99 | 99 | 99 | 98 | 97 |
| I | 92 | 73 | 81 | 84 | 85 | 88 | 87 | 100 | 100 | 100 | 100 | 100 | 100 | 100 | 100 |
| J | 100 | 99 | 100 | 98 | 99 | 95 | 100 | 100 | 100 | 100 | 100 | 100 | 100 | 100 | 100 |
| K | 89 | 99 | 96 | 82 | 97 | 82 | 91 | 99 | 98 | 99 | 98 | 99 | 99 | 89 | 92 |
| L | 100 | 100 | 100 | 100 | 100 | 100 | 100 | 100 | 100 | 100 | 100 | 100 | 100 | 100 | 100 |
| M | 93 | 95 | 93 | 92 | 86 | 75 | 88 | 100 | 100 | 100 | 100 | 100 | 100 | 100 | 100 |
| N | 93 | 65 | 74 | 61 | <50 | <50 | 56 | 100 | 100 | 100 | 98 | 98 | 98 | 100 | 100 |
| O | 100 | 94 | 96 | 97 | 92 | 93 | 96 | 100 | 100 | 100 | 100 | 100 | 100 | 100 | 100 |
| P | 92 | 74 | 75 | 70 | 65 | 63 | 70 | 100 | 100 | 100 | 100 | 100 | 100 | 100 | 100 |

^1^ Part. = Partitioning Scheme (single = one partition; BIC = Best partitioning scheme according to PartitionFinder Bayesian Information Criterion). BIC = 12S rDNA + 16S rDNA + Cytb codon 1 (GTR+G); Cytb codon 2 + COI codon 2 (HKY+G); Cytb codon 3 + COI codon 3 (GTR+G); COI codon 1 (GTR+G).

^2^ SH-like = approximate Likelihood Ratio Test (aLRT) for branch lengths with the Shimodaria-Hasegawa-like procedure.

^3^ Boot = Bootstrap search.

^4^ BrLens = Branch length Priors used in MrBayes v.3.2.2. DF = default. 0.5 and 10 refer to the prior value assigned to *β_T_* under the Gamma prior on tree length (*α_T_*, *α*, and *c,* were assigned a value of 1 in all cases; e.g. command “prset brlenspr=unconstrained:gammadir(1,0.1,1,1)”, where gammadir(*α_T_*,*β_T_*,*α*,*c*).

^5^ A polytomy prior was assumed in Phycas analyses.
