# Supplementary material for: Global Diversification at the Harsh Sea-Land Interface: Mitochondrial Phylogeny of the Supralittoral Isopod Genus Tylos (Tylidae, Oniscidea)
Source: PLoS One. 2014 Apr 15;9(4):e94081. doi: 10.1371/journal.pone.0094081 (PMC3988090; doi:10.1371/journal.pone.0094081)
Supplement: Table S4 — Percent Kimura-2-parameter distances for the concatenated dataset of four mitochondrial genes (MT; 1243 characters). (DOC) [file pone.0094081.s005.doc]

**Table S4. Percent Kimura-2-parameter distances for the concatenated dataset of four mitochondrial genes (MT; 1243 characters).**

Within and among *Tylos* species examined, and the outgroup (*Helleria*). Values on diagonal show range or maximum within-lineage divergence; number of taxa per clade indicated in parenthesis; otherwise only one taxon examined (na = not applicable).

|  | *Helleria brevicornis* | *T. spinulosus* | *T. chilensis* | *T. wegeneri* | *T. exiguus* | *T. maindroni* | *T. albidus* | *T. minor* | *T. opercularis* (2) | *T. granulifeus* (2) | *T. neozelanicus* | *T. europaeus* (2) | *T. ponticus* (3) | *T. granulatus* | *T. capensis* | *T. marcuzzi* (2) | *T. niveus* | *T. sp.* Yaguanabo | *T. punctatus* s. l*.* (48) |
| --- | --- | --- | --- | --- | --- | --- | --- | --- | --- | --- | --- | --- | --- | --- | --- | --- | --- | --- | --- |
| *T. spinulosus* | 30.5 | na |  |  |  |  |  |  |  |  |  |  |  |  |  |  |  |  |  |
| *T. chilensis* | 29.1 | 13.7 | na |  |  |  |  |  |  |  |  |  |  |  |  |  |  |  |  |
| *T. wegeneri* | 36.7 | 31.7 | 34.5 | na |  |  |  |  |  |  |  |  |  |  |  |  |  |  |  |
| *T. exiguus* | 33.6 | 28.8 | 31.5 | 35.1 | na |  |  |  |  |  |  |  |  |  |  |  |  |  |  |
| *T. maindroni* | 32.7 | 29.7 | 31.2 | 32.2 | 21.4 | na |  |  |  |  |  |  |  |  |  |  |  |  |  |
| *T. albidus* | 40.6 | 41.8 | 43 | 38.3 | 32.1 | 33.8 | na |  |  |  |  |  |  |  |  |  |  |  |  |
| *T. minor* | 26.1 | 26.4 | 29.7 | 35.5 | 28.1 | 25.9 | 34.4 | na |  |  |  |  |  |  |  |  |  |  |  |
| *T. opercularis* (2) | 32.4-36.4 | 33.9-38.7 | 32.9-40.2 | 32.2-39.5 | 27.1-33.3 | 27.7-30.3 | 30.7-33.3 | 14.9-18.9 | 14.4 |  |  |  |  |  |  |  |  |  |  |
| *T. granuliferus* (2) | 37-37.4 | 34.8-34.9 | 35.3-35.5 | 37.2-37.4 | 30.8-31 | 29.7-29.7 | 35.4-36.2 | 22.5-23.4 | 24.9-26.3 | 0.3 |  |  |  |  |  |  |  |  |  |
| *T. neozelanicus* | 33.7 | 32 | 32.9 | 33.3 | 28.6 | 26.2 | 34.9 | 31.5 | 26.1-32.6 | 29.7-29.7 | na |  |  |  |  |  |  |  |  |
| *T. europaeus* (2) | 32.4-33.8 | 30.7-31.9 | 31-31.2 | 35.6-39 | 24.8-26.8 | 22.8-25.5 | 34.2-36.5 | 23.3-25 | 25.4-32.9 | 29.3-30.2 | 24.7-27.4 | 3-5 |  |  |  |  |  |  |  |
| *T. ponticus* (3) | 34.8-36.2 | 32.7-33 | 32.9-33.3 | 36.8-37.6 | 22.5-24.9 | 24.3-26.9 | 34.7-36.5 | 23.5-24.3 | 27.7-33.7 | 30.1-32 | 27.7-28.3 | 15.1-17.1 | 4.2-14.8 |  |  |  |  |  |  |
| *T. granulatus* | 35.3 | 38.6 | 38.6 | 41.3 | 27 | 21.4 | 33.4 | 27 | 27-27.4 | 31.6-31.6 | 28.5 | 20.8-21.3 | 22.7-24.7 | na |  |  |  |  |  |
| *T. capensis* | 36.7 | 36.8 | 38.5 | 45.9 | 28.7 | 23.7 | 28.6 | 21.7 | 25.2-29.1 | 34.4-34.9 | 30.9 | 19.7-20.8 | 23.3-24.7 | 17.9 | na |  |  |  |  |
| *T. marcuzzi* (2) | 31.6-32.7 | 28.9-32.7 | 30.2-31.6 | 33.2-34.7 | 20.9-22.7 | 20.1-22 | 30.1-34.8 | 25.9-27.8 | 25.3-30.9 | 32.6-32.8 | 27-28.3 | 20.2-25.6 | 21.1-26.2 | 17.9-24.4 | 18.1-24.1 | 2.5 |  |  |  |
| *T. niveus* | 32.6 | 29.9 | 31.2 | 34.7 | 22.7 | 19 | 31.7 | 28 | 24.6-30.9 | 29.1-29.1 | 25.4 | 23.6-25.9 | 24.8-26.3 | 22 | 25.1 | 16.5-20.5 | na |  |  |
| *T. sp.* Yaguanabo | 32.9 | 30.5 | 30.8 | 33.9 | 21.4 | 21.9 | 31.6 | 27.8 | 25.2-29.2 | 30.9-30.9 | 27 | 24.1-25.4 | 24.9-26.2 | 22.9 | 25.2 | 14.9-18.8 | 14.1 | na |  |
| *T. punctatus* s. l. (48) | 24.4-38.2 | 21.4-30.6 | 22.2-31.9 | 27.6-36.2 | 17.4-23.7 | 18.1-24.6 | 33.2-40.5 | 20.8-31.4 | 23.4-34.8 | 28.5-32.3 | 23.2-30.4 | 13.6-26.3 | 14.7-26.7 | 14.9-26.9 | 16.4-29.4 | 14.9-23.5 | 9.5-18.4 | 11.5-17.8 | 16.4 |
|  |  |  |  |  |  |  |  |  |  |  |  |  |  |  |  |  |  |  |  |
