# Supplementary material for: Global Diversification at the Harsh Sea-Land Interface: Mitochondrial Phylogeny of the Supralittoral Isopod Genus Tylos (Tylidae, Oniscidea)
Source: PLoS One. 2014 Apr 15;9(4):e94081. doi: 10.1371/journal.pone.0094081 (PMC3988090; doi:10.1371/journal.pone.0094081)
Supplement: Table S5 — Percent Kimura-2-parameter distances for the COI gene (600 characters). (DOC) [file pone.0094081.s006.doc]

**Table S5. Percent Kimura-2-parameter distances for the COI gene (600 characters).**

Within and among *Tylos* species examined, and the outgroup (*Helleria*). Values on diagonal show range or maximum within-lineages divergence; number of taxa per clade indicated in parenthesis; otherwise only one taxon examined (na = not applicable).

|  | *Helleria brevicornis* | *T. spinulosus* | *T. chilensis* | *T. wegeneri* | *T. exiguus* | *T. maindroni* | *T. opercularis* Australia | *T. granulifeus* (2) | *T. neozelanicus* | *T. ponticus* (3) | *T. europaeus* (2) | *T. marcuzzi* (2) | *T. niveus* | *T. sp.* Yaguanabo | *T. punctatus* s.l. (48) |
| --- | --- | --- | --- | --- | --- | --- | --- | --- | --- | --- | --- | --- | --- | --- | --- |
| *T. spinulosus* | 26.4 | na |  |  |  |  |  |  |  |  |  |  |  |  |  |
| *T. chilensis* | 25.5 | 13.4 | na |  |  |  |  |  |  |  |  |  |  |  |  |
| *T. wegeneri* | 29.3 | 25.4 | 26.7 | na |  |  |  |  |  |  |  |  |  |  |  |
| *T. exiguus* | 28.4 | 22.6 | 24.3 | 27.9 | na |  |  |  |  |  |  |  |  |  |  |
| *T. maindroni* | 27.8 | 22.9 | 25.6 | 25.5 | 22.3 | na |  |  |  |  |  |  |  |  |  |
| *T. opercularis* Australia | 28.4 | 31 | 29 | 26.5 | 23.5 | 25.7 | na |  |  |  |  |  |  |  |  |
| *T. granuliferus* (2) | 30.6 | 31.1 | 31.5 | 29.2 | 27.5 | 30.4 | 24.4 | 0 |  |  |  |  |  |  |  |
| *T. neozelanicus* | 24.1 | 26.7 | 24.8 | 25.3 | 21.8 | 22.2 | 23.6 | 28.3 | na |  |  |  |  |  |  |
| *T. ponticus* (3) | 27.2-29.6 | 25.5-26.9 | 26.1-27.8 | 28.2-29.2 | 18.8-23 | 24.2-27.7 | 24.4-26.4 | 27-29.7 | 23.3-24.7 | 5.3-17.3 |  |  |  |  |  |
| *T. europaeus* (2) | 22.8-29.1 | 21.1-24.9 | 20-25.7 | 26.1-26.5 | 19-24.8 | 20.9-26.8 | 22.8-24.9 | 25.8-28.1 | 19.9-22.7 | 16.7-20.4 | 5.3-7.2 |  |  |  |  |
| *T. marcuzzi* (2) | 25.9-26.1 | 23.8-26.6 | 24.1-24.5 | 25.1-25.5 | 21.7-23.1 | 21.6-22.5 | 25.6-27.3 | 32-33.9 | 24.2-24.7 | 22.1-26.1 | 24.5-25.8 | 3.2 |  |  |  |
| *T. niveus* | 25.4 | 22.5 | 24.8 | 26.6 | 23.3 | 18.4 | 19.8 | 27.2 | 19.6 | 24.6-25.4 | 21.2-23.5 | 19.4-20.2 | na |  |  |
| *T. sp.* Yaguanabo | 25.2 | 23.5 | 24.2 | 27.5 | 21.8 | 20.9 | 22.9 | 32.1 | 22.9 | 23.1-24.6 | 20.9-25.2 | 16.4-18.0 | 16 | na |  |
| *T. punctatus* s.l. (48) | 20.8-26.3 | 20.5-23.7 | 22.2-28.2 | 25-29 | 16.9-22.8 | 17.5-23.8 | 21.3-29 | 26.4-31.6 | 19-25.9 | 18.2-26.8 | 17.3-25.3 | 15.9-22.6 | 13.6-19 | 15.3-19 | 17.2 |
|  |  |  |  |  |  |  |  |  |  |  |  |  |  |  |  |
